# Genome-wide characterization and expression analysis of the GATA transcription factor family in response to salt and drought stress in barley (*Hordeum vulgare* L.)

**DOI:** 10.3389/fpls.2025.1661591

**Published:** 2025-09-17

**Authors:** Haitao Zhang, Peng Yu, Hongwu Xiao, Chengyu Wang, Xiaofeng Yan, Xiaoqin Zhang

**Affiliations:** ^1^ College of Agronomy, Anhui Agricultural University, Hefei, China; ^2^ Agricultural and Rural Bureau of Feixi County, Hefei, China; ^3^ College of Life and Environmental Sciences, Hangzhou Normal University, Hangzhou, China

**Keywords:** barley, GATA transcription factor, bioinformatics, salt and drought stress, expression analysis

## Abstract

GATA transcription factors play a crucial role in regulating plant growth and development as well as stress responses. However, systematic analysis of *GATA* genes in barley remains uncharacterized, and their functional roles in salt and drought stress responses are poorly understood. This study conducted genome-wide identification and gene expression analysis of the GATA transcription factor family in barley through bioinformatics approaches. A total of 27 *HvGATA* genes were identified and divided into 4 subfamilies (I-IV), which were unevenly distributed on 7 chromosomes. Overall tertiary structural similarity of GATA proteins existed with differences, but similarity within the same subfamily was higher. Fragment repetition has been identified as the main driving factor for the expansion of the *HvGATA* family. Members of the same subfamily exhibit highly conserved exon-intron structures and motif compositions, indicating strong functional conservation among subfamilies. *Cis*-element analysis of the *HvGATA* promoter reveals potential regulatory complexity and enrichment of stress-responsive elements related to hormone signals (such as abscisic acid) and drought stress responses. qRT-PCR analysis showed that at 6 h after salt treatment, *HvGATA1*, *HvGATA19*, and *HvGATA25*, were significantly up-regulated (P < 0.05). Under drought treatment, at least three *HvGATA* genes (*HvGATA1*, *HvGATA19*, and *HvGATA25*) showed dynamic expression patterns, which were down-regulated and up-regulated at 2 h, 24 h, and 12 h, respectively, indicating that they have potential roles in regulating salt tolerance and drought tolerance of barley. This research provides new insights into the molecular mechanisms of salt and drought tolerance in barley and offers potential targets for enhancing the crop’s stress resistance under abiotic stresses.

## 1 Introduction

Transcription factors (TFs) play indispensable roles in plant growth and development, regulating processes such as cell differentiation (Stevanovic et al., 2022), physiological metabolism (Rueda-Lopez et al., 2015), signal transduction (Liu et al., 2022), stress responses (Darigh et al., 2022; Zha et al., 2022), and other biological functions. 64 transcription factor families have been identified in plants (Guo et al., 2008), including *WRKY* (Rushton et al., 2010; Jiang et al., 2017), *MYB* (Dubos et al., 2010), *GRAS* (Zeng et al., 2019), *bHLH* (Wang et al., 2018), and *GATA* (Kim, 2024). Among them, GATA is a transcription factor widely present in eukaryotes and an important regulatory protein that specifically binds to the DNA sequence WGATAR (W is T or A, R is G or A), thereby regulating the expression of downstream genes (Lowry and Atchley, 2000; Scazzocchio, 2000) and modulating plant growth and development. For instance, it plays a crucial role in regulating chlorophyll synthesis (Hudson et al., 2011) and stress responses (Huang et al., 2009). The GATA protein domain contains a class IV zinc finger structure characterized by CX_2_CX_17_-_20_CX_2_C, followed by a conserved region. Some members of this zinc finger structure can specifically bind to the DNA sequence (A/T) GATA (A/G) in the gene regulatory region, thereby modulating gene transcription level (Lowry and Atchley, 2000; Scazzocchio, 2000). Most GATA transcription factors in fungi and animals contain zinc finger domains characterized by CX_2_CX_17_CX_2_C or CX_2_CX_18_CX_2_C (Teakle and Gilmartin, 1998; Scazzocchio, 2000), while plant GATA transcription factors typically contain 18–20 residues. The zinc finger domain in plants is typically CX_2_CX_18_CX_2_C or CX_2_CX_20_CX_2_C, and it contains a single zinc finger motif (Lowry and Atchley, 2000; Behringer and Schwechheimer, 2015).

The GATA transcription factor *NTL1*, cloned from tobacco firstly, has been reported to participate in nitrogen metabolism pathways (Daniel-Vedele and Caboche, 1993). In plants, GATA transcription factors play crucial regulatory roles in nitrogen metabolism, stress responses, growth, development, and hormone signaling pathways (Richter et al., 2013). In *Arabidopsis thaliana*, GATA transcription factor family members, *GNC* and its homologous gene *GNLI*, have been shown to involve in chlorophyll biosynthesis, signal transduction, and regulation of developmental processes. Additionally, during plant growth, *GNC* and *GNL* regulate brassinosteroids, while *GATA2* regulates brassinosteroids during *Arabidopsis* photomorphogenesis (Luo et al., 2010; Richter et al., 2013). In maize, GATA transcription factor family members *GATA7* and *GATA33* regulate the growth and development of the shoot apical meristem (Zhan et al., 2015). In rice, the GATA transcription factor family members *NECKLEAF1* (Wang et al., 2009) and *Cga1* (Hudson et al., 2013) have been found to regulate rice organ formation, chlorophyll synthesis, and chloroplast development. In wheat (*Triticum aestivum*), the transcription factor TaGATA1 enhances seed dormancy by directly regulating *TaABI5* expression, thereby improving resistance to pre-harvest sprouting (Wei et al., 2023). To date, genome-wide analyses of GATA transcription factor families have been conducted in various crops. For instance, 30, 28, and 33 members have been identified in *Arabidopsis thaliana* (Manfield et al., 2007), *Oryza sativa* (Gupta et al., 2017), and *Sorghum bicolor* (Yao et al., 2023), respectively, providing valuable insights into their structure and biological functions in other crops.

Barley (*Hordeum vulgare* L.) is the fourth most important cereal crop globally, cultivated extensively across diverse agroecological systems. Barley is rich in protein, vitamins, trace elements, and dietary fiber and exhibits agronomically valuable traits, including a short growth cycle, high tolerance to drought and cold, and broad adaptability (Geng et al., 2022). The complete genome sequence of barley has been fully established, enabling comprehensive molecular biology studies on this species. However, systematic identification of barley *GATA* family genes and their functional roles in abiotic stress responses remains limited. In this study, systematically identified barley *GATA* family genes through bioinformatics approaches and analyzed their phylogeny, gene structure, conserved motifs, *cis*-acting elements, and chromosomal distribution. Furthermore, putative orthologs of barley *GATA* family genes in *Arabidopsis* and rice were identified for collinearity analysis to infer evolutionary relationships. Additionally, the barley cultivar Morex were subjected to salt stress (150 mM NaCl) and drought stress (20% PEG-6000) to evaluate abiotic stress responses. The transcriptional levels of four *HvGATA* genes were quantified using quantitative real-time PCR to abiotic stress provide insights into the functional characterization of barley GATA transcription factors.

## 2 Materials and methods

### 2.1 Identification of *GATA* members in barley

To identify members of the barley *GATA* family, the whole genome sequence, protein sequence, and gene annotation file of barley were obtained from the publicly released Ensembl Plant database (https://plants.ensembl.org/Hordeum_vulgare/Info/Index), and protein sequences of *Arabidopsis* and rice were retrieved from previously published data (Reyes et al., 2004). Putative HvGATA proteins were identified by performing a BLASTp search against the barley proteome (score ≥100, E-value ≤1e−10) (Altschul et al., 1997). Subsequently, the Pfam database (https://www.ebi.ac.uk/Tools/hmmer/) was used to screen candidate proteins for the presence of the GATA zinc finger domain (PF00320) using its corresponding hidden Markov model (HMM) profile with a significance threshold of 0.01 (Finn et al., 2008, 2011). To further validate conserved domains, CD-Search (https://www.ncbi.nlm.nih.gov/Structure/cdd/cdd.shtml) and SMART (http://smart.emblheidelberg.de/) were employed (Letunic and Bork, 2018; Yang et al., 2020). Proteins lacking the GATA domain were excluded from further analysis.

### 2.2 Bioinformatics analysis of HvGATA members

ExPASy (https://web.expasy.org/) was used to predict the amino acid length (aa), molecular weight (MW), isoelectric point (pI), instability index (II), aliphatic index, and grand average of hydropathicity (GRAVY). And use BUSCA (http://busca.biocomp.unibo.it/) for subcellular localization prediction.

### 2.3 Phylogenetic analysis of HvGATA proteins

Based on studies of *GATA* family genes in *Arabidopsis* and rice (Bi et al., 2005; He et al., 2018), zinc finger domain sequences of 30 *Arabidopsis* and 28 rice GATA proteins were downloaded. Multiple sequence alignment of barley, *Arabidopsis*, and rice GATA proteins was performed using Clustal W (Larkin et al., 2007) with default parameters in MEGA11 (Tamura et al., 2021). A phylogenetic tree was constructed using the maximum likelihood method with 1,000 bootstrap replicates.

### 2.4 Analysis of conserved motifs, gene structures, and *Cis*-acting elements

Conserved domains in barley GATA proteins were analyzed with DNAMAN. The MEME suite (https://meme-suite.org/meme/tools/meme) was employed to identify protein motifs in the full-length sequences of 27 *HvGATAs* (Bailey et al., 2009). Gene structures of *HvGATAs* were visualized using TBtools by aligning coding sequences (CDS) with their corresponding genomic DNA sequence (Chen et al., 2020). *Cis*-regulatory elements in the promoter regions (2,000 bp upstream) of *HvGATA* genes were predicted using PlantCare (http://bioinformatics.psb.ugent.be/webtools/plantcare/html/).

### 2.5 Detection and homology analysis of paralogous gene pairs

Homologous gene pairs and syntenic relationships in barley *GATA* genes were identified using the Multiple Collinearity Scan toolkit (MCScanX) with default parameters (Wang et al., 2012). To predict the function of barley *GATA* family genes, syntenic relationships of homologous *GATA* genes among barley, *Arabidopsis*, and rice were analyzed. Circos software (Krzywinski et al., 2009) was utilized to visualize the syntenic relationships of *GATA* genes across these three species.

### 2.6 Analysis of *HvGATA* genes expression profiles in barley transcriptome data

The expression profiles of barley *GATA* genes in different tissues and under salt/drought stress conditions were obtained from the BarleyExpDB database (http://barleyexp.com/index.html). These data were visualized using TBtools software to generate expression heatmaps of barley *GATA* genes.

### 2.7 Plant materials, stress treatment, and qRT-PCR analysis of 4 *HvGATA* genes

The barley variety Morex was used in this study. Seeds were placed on filter paper in germination boxes, germinated in darkness at 24°C for 48 h, transferred to Hoagland medium in 96-well plates, and cultivated in a growth chamber at 24°C (day) and 18°C (night) with a 14 h/10 h day/night cycle. When seedlings reached the three-leaf stage, they were subjected to two abiotic stress treatments, salt stress (150 mM NaCl) and drought stress (20% PEG-6000) (Zheng et al., 2021). Three biological replicates were performed for each sample, and leaves were collected at 0, 2, 6, 12, and 24 h following the initiation of each stress treatment. All samples were frozen in liquid nitrogen and stored at -80°C for RNA extraction.

Total RNA was extracted using the SteadyPure Plant RNA Extraction Kit (Accurate Biotechnology Co., Ltd., Hunan, China). RNA integrity was verified by 1% agarose gel electrophoresis, and concentration was measured using a UV-Vis spectrophotometer. First-strand cDNA was synthesized from total RNA with the Evo M-MLV RT Premix (Accurate Biotechnology, Hunan, China) for qPCR. Gene-specific primers for four *HvGATA* genes were designed with Primer 5.0 software (**Supplementary Table S1**), yielding amplicons of 80–150 bp. *HvActin* (HORVU6Hr1G054520) was used as the internal reference gene. qRT-PCR was performed on a Roche LightCycler 96 system using the SYBR Green Premix Pro Taq HS qPCR Kit (Accurate Biotechnology Co., Ltd., Hunan, China) with the following reaction components: 1.0 µL cDNA, 10.0 µL 2× SYBR mixture, 0.8 µL primer mix (forward/reverse), and 8.2 µL ddH_2_O. Thermal cycling conditions were 95°C for 3 min; 40 cycles of 95°C for 5 s and 58°C for 30 s. Gene expression levels were calculated using the 2^−ΔΔCT^ method.

### 2.8 Prediction of structural features and protein interaction networks in *HvGATA* transcription factors

Secondary structure prediction for barley GATA proteins was performed using SOPMA (https://npsa.lyon.inserm.fr/cgi-bin/npsa_automat.pl?page=/NPSA/npsa_sopma.html). Tertiary structure modeling of identified GATA protein sequences was conducted via the SWISS-MODEL web server (https://swissmodel.expasy.org/interactive/). Protein-protein interaction (PPI) network analysis of barley GATA proteins was executed using the STRING database (https://cn.string-db.org/).

## 3 Results

### 3.1 Identification and physicochemical properties of HvGATA members

The hidden Markov model (HMM) of the GATA domain andthe BLASTp method were employed to identify 27 *HvGATA* genes in the barley genome, designated as *HvGATA1*-*HvGATA27* according to their chromosomal locations (**Supplementary Table S2**). The encoded proteins ranged from 155 to 775 amino acids in length, with molecular weights ranging from 17343.27 Da to 88608.50 Da. Their isoelectric points varied from 4.66 to 10.22, with 13 members classified as acidic (pI < 7) and 14 as basic (pI > 7), indicating a mixture of acidic and basic proteins. Instability coefficient analysis showed that 26 HvGATA proteins were unstable (values > 40), with HvGATA21 protein exhibiting the highest instability coefficient (86.57). In contrast, HvGATA27 protein was relatively stable, with an instability index below 40. All HvGATA proteins displayed negative mean hydrophobicity coefficients, confirming their hydrophilic nature, though the extent of HvGATA proteins was localized to the nucleus, 3 to extracellular space, and HvGATA7 protein and HvGATA24 protein to chloroplasts.

### 3.2 Phylogeny, classification, and multiple sequence alignment of HvGATA proteins

**Figure 1:**
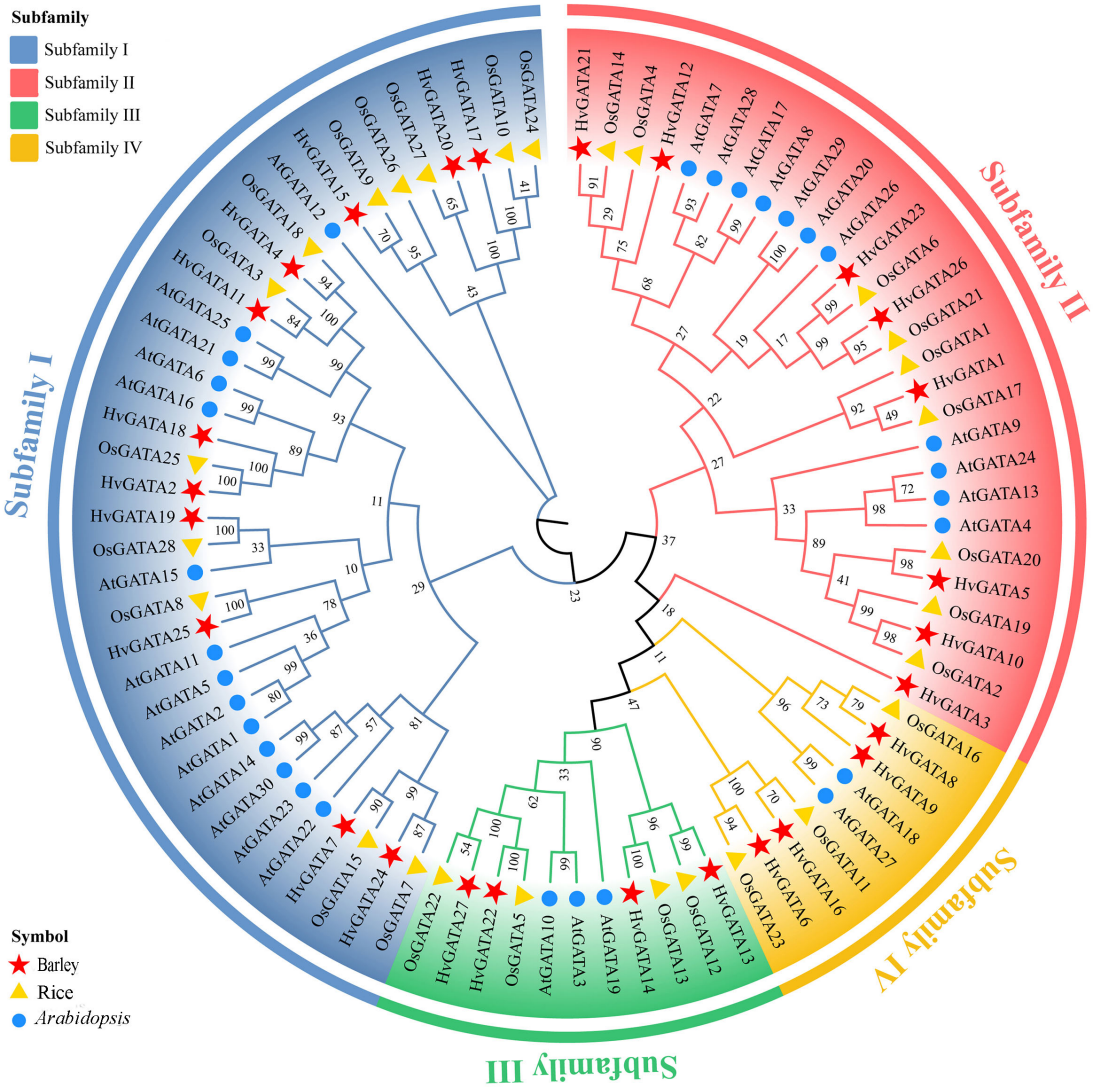
Phylogenetic tree of GATA proteins from barley, rice, and Arabidopsis. The MEGA 11 Maximum Likelihood (ML) method with 1,000 bootstrap replicates was used to generate the phylogenetic tree. Four subfamilies were categorized and indicated with colors.

Using MEGA11, a phylogenetic tree was constructed from 27 HvGATA, 28 OsGATA, and 30 AtGATA proteins (**Figure 1**, **Supplementary Table S3**). The analysis revealed four subfamilies. Subfamily I contained the highest number of *HvGATA* members (11), followed by Subfamily II (8), while Subfamilies III and IV each comprised four members, respectively. 10 HvGATA proteins clustered closely with AtGATA protein and OsGATA protein orthologs, suggesting conserved functions through evolution.

**Figure 2:**
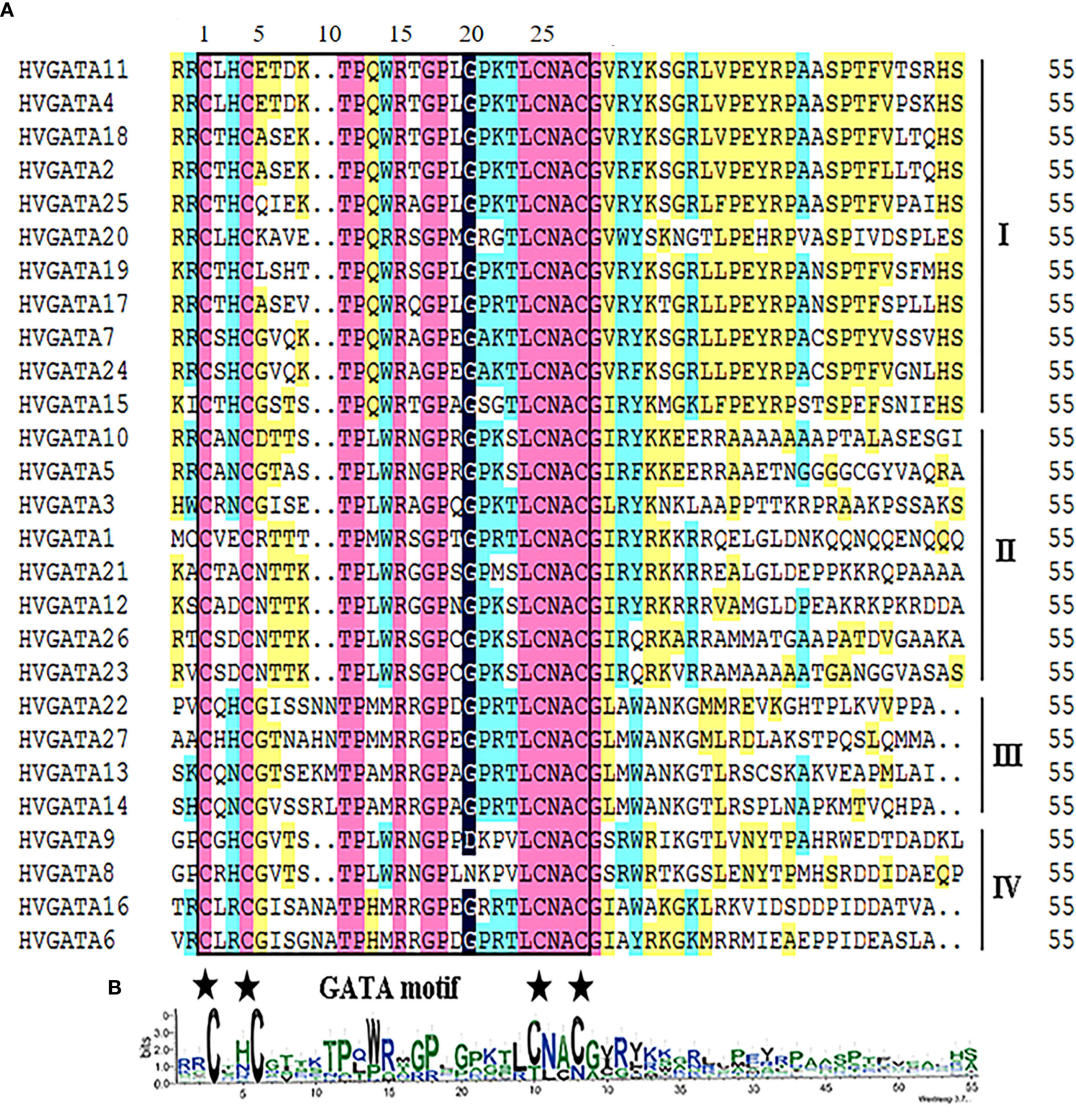
Amino acid sequence alignment of HvGATAs. **(A)** GATA motifs and amino acid locations are marked with boxes and asterisks; **(B)** the sequence logo of the GATA pattern is displayed at the bottom.

Multiple sequence alignment of conserved regions (**Figure 2**) indicated that all barley GATA proteins harbor a single plant-specific GATA domain, Consistent with rice and *Arabidopsis*, 21 HvGATA proteins (from Subfamilies I, II, and IV) displayed the motif CX_2_CX_18_CX_2_C, whereas 6 HvGATA proteins (four from Subfamily III and two from Subfamily IV) featured an extended variant (CX_2_CX_20_CX_2_C). All subfamilies shared conserved sequences (e.g., T-7, P-8, G-13, P-14), highlighting both conservation and diversification within GATA domains.

### 3.3 Conserved motifs, gene structures, and *Cis*-acting elements of *HvGATA* genes

**Figure 3:**
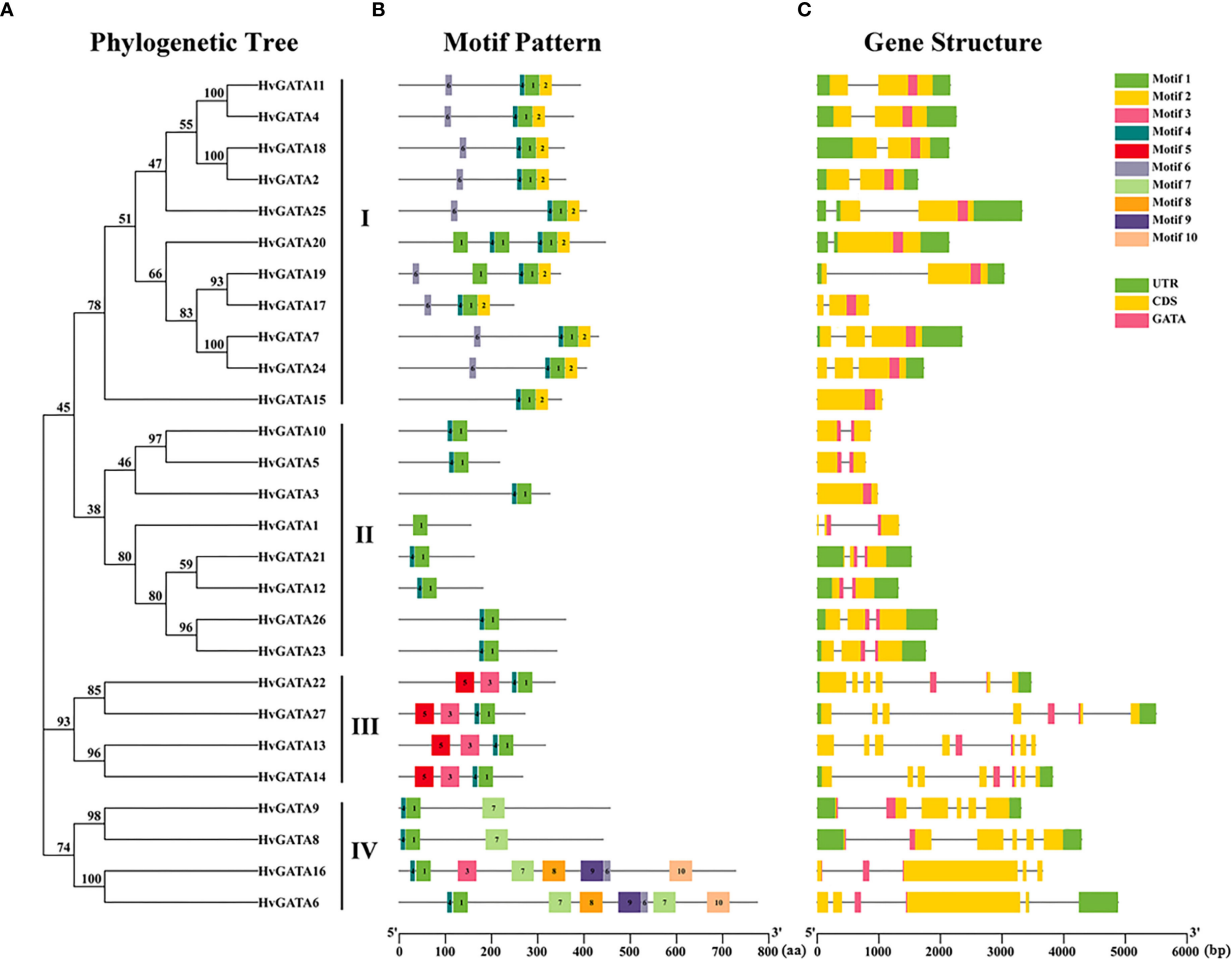
Conserved motif and gene structure analysis of *HvGATAs*. **(A)** The Maximum Likelihood (ML) method with 1,000 bootstrap replicates was used to generate the phylogenetic tree. **(B)** The 1–10 amino acid motifs in the HvGATAs protein are represented by 10 different colored boxes, with black lines indicating relative protein lengths. **(C)** UTR (untranslated region), CDS (coding sequence), GATA domains, and introns are represented by green squares, yellow squares, pink squares, and black lines, respectively. The sizes of exons and introns are estimated using the scale line at the bottom.

TBtools was used to construct a phylogenetic tree of 27 HvGATA proteins (**Figure 3A**), while the MEME online program was employed to analyze their conserved motifs (**Figure 3B**, **Supplementary Table S4**). Analysis revealed that the number of motifs among *HvGATA* members ranged from 1 to 8. Specifically, *HvGATA1* contained the lowest motifs, whereas *HvGATA6* and *HvGATA16* had the highest number (8 motifs each). Notably, Motif 1 was conserved across all barley GATA proteins, suggesting its critical role in the evolution of this gene family. Subgroup-specific patterns were observed: Motif 2 and Motif 6 were exclusive to Subfamily I; Motif 4 was absent solely in *HvGATA1*; Motif 5 was restricted to Subfamily III; and Motif 7 was unique to Subfamily IV. Additionally, *HvGATA6* and *HvGATA16* uniquely contained Motifs 8-10, while *HvGATA16* shared Motif 3 with all Subfamily III members. The widespread conservation of motifs among most *HvGATA* members implies functional similarities within this gene family.

Gene structure analysis (**Figure 3C**), revealed that *HvGATA* genes contain 0–7 introns. Among the 27 genes, two (*HvGATA3* and *HvGATA18*) lacked introns, 10 harbored a single intron, seven contained two introns, one exhibited four introns, three possessed five introns, and two displayed six or seven introns, respectively. Subfamilies I and II exhibited minimal intron content (0–2 introns). In contrast, Subfamilies III and IV showed marked intron expansion: Subfamily III genes contained 6–7 introns, while Subfamily IV genes had 4–5 introns. These results highlight a significant increase in intron number in Subfamilies III and IV compared to Subfamilies I and II, suggesting divergent evolutionary dynamics among subfamilies.

**Figure 4:**
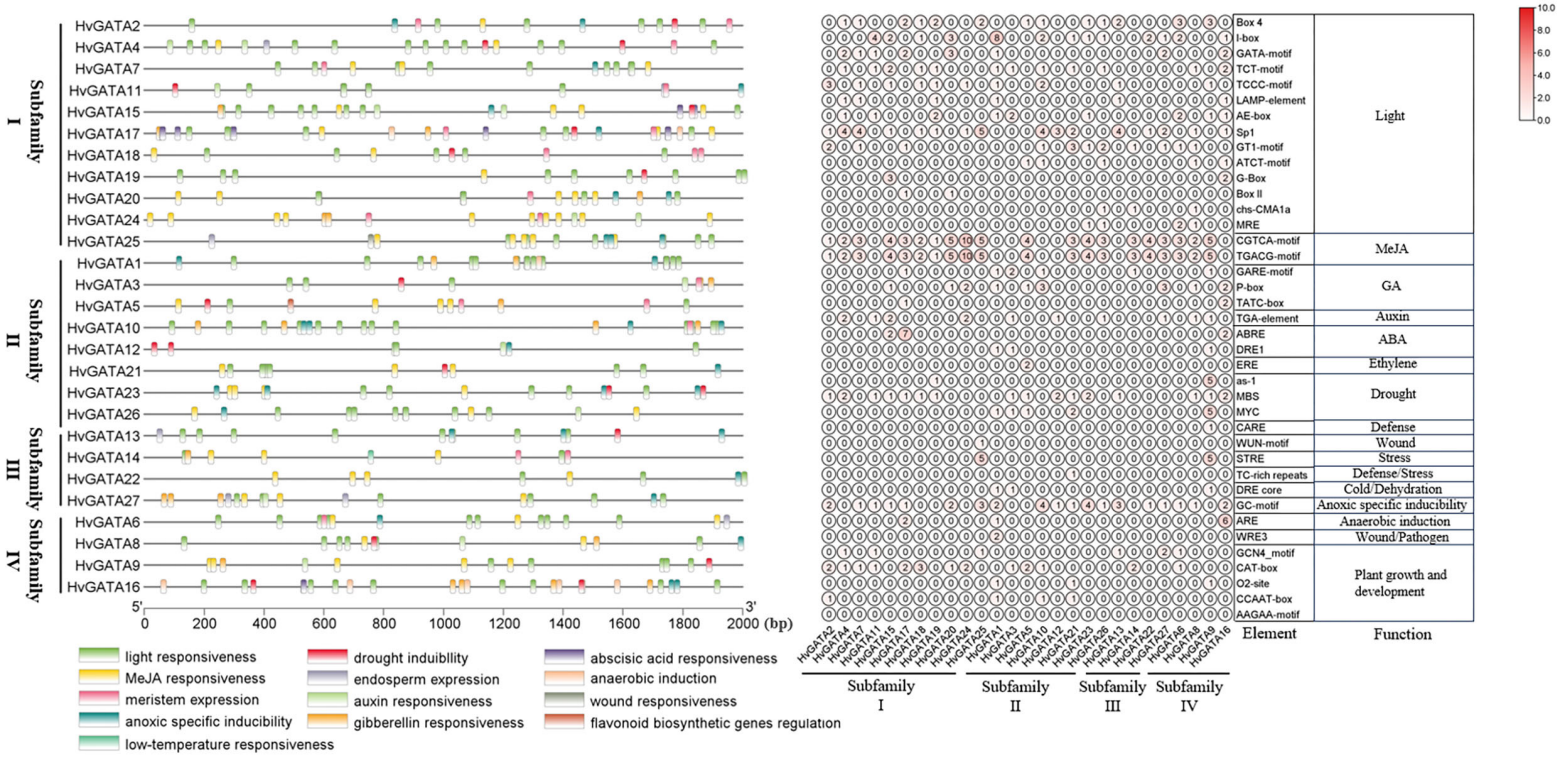
*Cis*-acting element of the promoter region (upstream 2,000 bp) of *HvGATA* genes. Various types of *cis*-elements and their respective locations in each *HvGATA* gene. The numbers of different *cis*-acting elements in the initiation regions of *HvGATA* genes. Each *cis*-acting element and function was shown on the right, and the corresponding number of them was indicated by the color scale.

To investigate the regulatory potential of promoters in the barley *HvGATA* gene family, *cis*-acting elements were computationally identified within the 2,000 bp upstream promoter regions of 27 *HvGATA* genes (**Figure 4**, **Supplementary Table S5**). Photoresponsive elements (187 total) and methyl jasmonate (MeJA)-responsive elements (140 total) were the most abundant, with Sp1 (36 occurrences) and I-box (29 occurrences) dominating the light-responsive category. Among hormone-related elements, MeJA-responsive motifs (CGTCA-motif and TGACG-motif) were present in 20 promoters, followed by gibberellin (GA)-responsive elements (31 total). Notably, 26 promoters harbored multiple hormone-responsive motifs, including MeJA, abscisic acid (ABA; ABRE, DRE1), auxin (IAA; TGA-element), and GA-responsive elements (GARE-motif, P-box, TATC-box), while *HvGATA13* lacked these motifs.

Stress-responsive *cis*-elements were also identified: drought-related motifs (as-1, MYC, and MBS) totaled 36, with as-1 restricted to *HvGATA9* and *HvGATA19*, MYC to *HvGATA1*, *HvGATA3*, *HvGATA5*, *HvGATA9*, and *HvGATA21*; and MBS present in all 16 promoters analyzed. Additionally, 12 defense/stress motifs (e.g., STRE, CARE, and TC-rich repeats) were detected, with STRE in *HvGATA9* and *HvGATA25*; and CARE in a single promoter. Elements linked to hypoxia (ARE, GC-motif), pathogens (WRE3, WUN), and drought (DRE core) occurred in 3, 1, and 19 promoters, respectively. These findings suggest that *HvGATA* genes may regulate not only plant growth but also hormone signaling and abiotic stress responses, particularly drought adaptation.

### 3.4 Chromosome distribution, gene replication, and collinearity analysis of *HvGATA* genes

**Figure 5:**
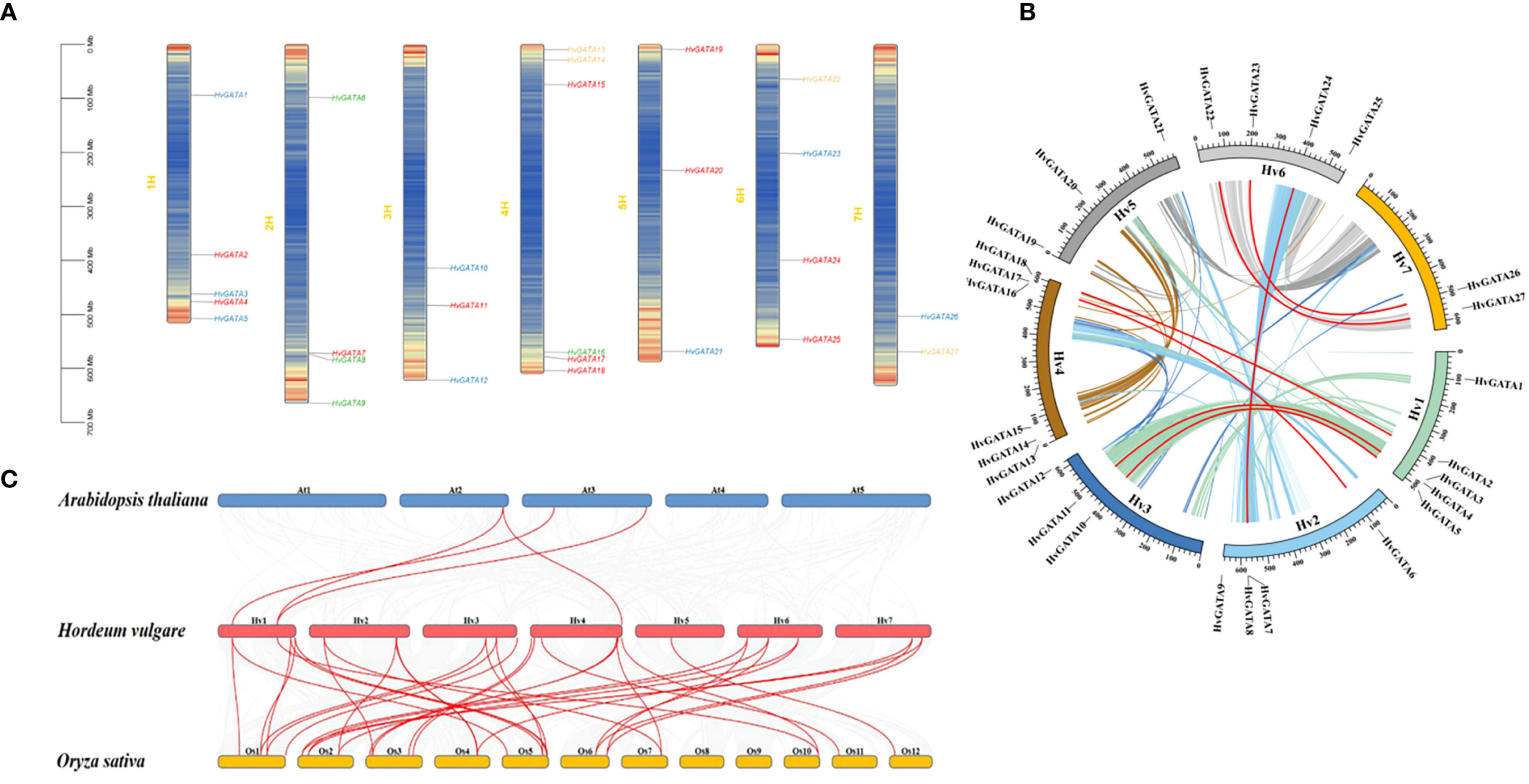
**(A)** The distribution of *HvGATA* genes on chromosomes. The four HvGATA subfamily members are represented by red, blue, orange, and green, respectively; each corresponding chromosome number is labeled on its left, the 0–700 Mb scale on the left represents the chromosome length, and the gene density is displayed in color depth. **(B)** Chromosome distribution and gene duplication relationship of *HvGATA genes*. The colored lines represent collinear regions in the barley genome, and the red lines represent large segments of replicated *HvGATA* gene pairs. **(C)** Synteny analysis of *GATAs* in *Hordeum vulgare*, *Arabidopsis thaliana*, and *Oryza sativa*. The red lines represent syntenic *GATA* gene pairs. The numbers on the chromosomes indicate chromosome numbers.

To elucidate the chromosomal distribution of *GATA* genes in barley, 27 *HvGATA* genes were mapped to seven chromosomes (**Figure 5A**). The distribution frequency varied significantly across chromosomes: Chromosome 1 harbored the highest number of genes (5 genes, 18.52%), followed by Chromosome 4 (6 genes, 22.22%). Chromosomes 2 and 6 each contained 4 genes (14.81% per chromosome), while Chromosomes 3, 5, and 7 showed lower densities with 3 genes (11.11%) on Chromosome 3 (*HvGATA10*-*HvGATA12*), 3 genes (11.11%) on Chromosome 5 (*HvGATA19*-*HvGATA21*), and 2 genes (7.41%) on Chromosome 7 (*HvGATA26* and *HvGATA27*).

MCScanX based segmental duplication analysis of the 27 *HvGATA* genes identified seven duplication events involving 14 genes (**Figure 5B**, **Supplementary Table S6**), accounting for 51.85% (14/27) of the gene family. These duplicated pairs included *HvGATA2*/*HvGATA18*, *HvGATA4*/*HvGATA11*, *HvGATA5*/*HvGATA10*, *HvGATA6*/*HvGATA16*, *HvGATA7*/*HvGATA24*, *HvGATA22*/*HvGATA27*, and *HvGATA23*/*HvGATA26*. Subfamily classification revealed that four duplication pairs (57.14%) belonged to Subfamily I, two pairs (28.57%) to Subfamily II, and one pair each (14.29%) to Subfamilies III and IV. This uneven distribution suggests that segmental duplications may have been a major driver of *HvGATA* gene family expansion. Furthermore, *HvGATA* genes were asymmetrically distributed across seven barley linkage groups (LGs). LG1 and LG6 harbored the highest number of duplicated genes (3 genes each, 21.43% of total duplications), followed by LG2, LG3, LG4, and LG7 (2 genes each, 14.29% per LG).

To elucidate the evolutionary dynamics of *HvGATA* genes, synteny analysis was conducted between two monocotyledonous species (barley and rice) and one dicotyledonous species (*Arabidopsis*). The results revealed (**Figure 5C**, **Supplementary Table S7**), 23 *OsGATA* genes exhibiting orthologous relationships with 21 *HvGATA* genes, while 3 *AtGATA* genes showed orthology with 3 *HvGATA* genes. These findings indicate that barley, as a monocotyledonous poaceae species, shares a closer phylogenetic relationship with rice than with the dicotyledonous *Arabidopsis*. Furthermore, collinear gene pairs are often functionally conserved, suggesting that the 33 barley-rice collinear *GATA* gene pairs and 4 barley-*Arabidopsis* collinear *GATA* gene pairs may perform analogous roles in their respective species. This syntenic conservation underscores potential functional similarities driven by shared evolutionary origins.

### 3.5 Expression profile of *HvGATA* genes in different tissues and abiotic stresses

**Figure 6:**
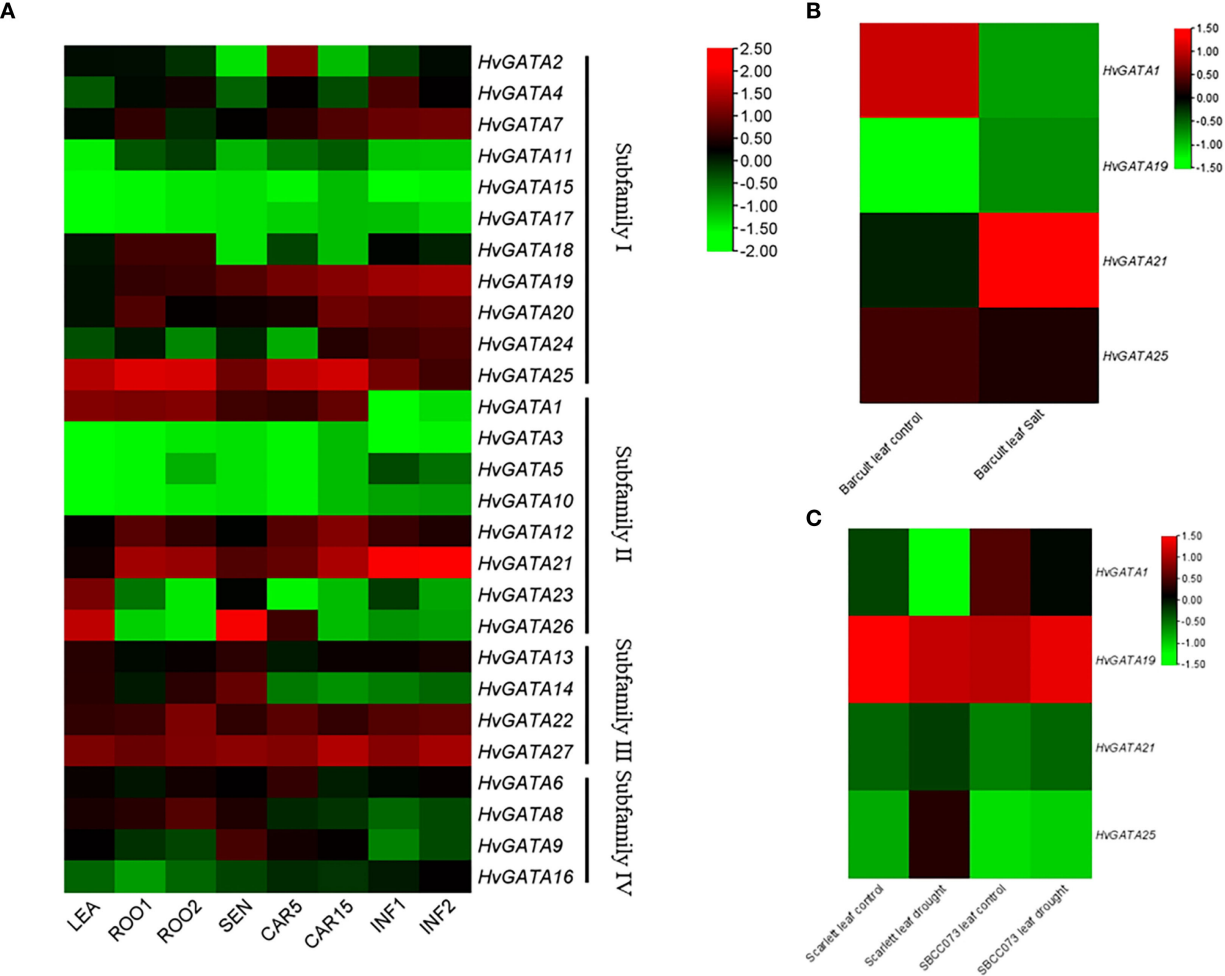
Expression profiles analysis of *HvGATA* genes across different tissues and salt/drought stress treatments. Different colors in the figure represent gene expression levels, with green indicating low gene expression and red indicating high gene expression. **(A)** The color scale represents the relative expression level from high (red) to low (green). CAR15 (seed 15 days after fertilization); LEA (10cm bud); ROO1 (root of 10 cm seedling); ROO2 (28-day root); SEN (senescent leaf); INF2 (1-1.5 cm inflorescence); INF1 (5 mm inflorescence); CAR5 (seed 5 days after fertilization). **(B)** Scarlett leaf control refers to the expression level of barley variety Scarlett leaves under normal treatment. Scarlett leaf drought refers to the expression of leaves of barley variety Scarlett under drought treatment. SBC073D leaf control was the expression level of barley leaves variety SBC073D under normal treatment. SBC073D leaf drought refers to the expression of barley leaf SBC073D under drought treatment. **(C)** Barcult leaf control is the expression level of leaves under normal treatment. Barcult leaf Salt is the expression of leaves under salt treatment.

To investigate the tissue-specific expression patterns of *HvGATA* genes, transcript levels of 27 *HvGATA* genes were analyzed across eight barley tissues (**Figure 6A**, **Supplementary Table S8**). 15 genes (*HvGATA1*, *HvGATA2*, *HvGATA4*, *HvGATA6-HvGATA9*, *HvGATA12*, *HvGATA13*, *HvGATA19*-*HvGATA22*, *HvGATA25*, and *HvGATA27*) exhibiting ubiquitous and/or high expression across multiple tissues, suggesting their potential roles in barley growth and development, 6 genes (*HvGATA3*, *HvGATA5*, *HvGATA10*, *HvGATA11*, *HvGATA15*, *HvGATA17*) with low or undetectable expression in all tested tissues, and 6 genes (*HvGATA14*, *HvGATA16*, *HvGATA18*, *HvGATA23*, *HvGATA24*, *HvGATA26*) showing preferential expression in specific tissues. For instance, *HvGATA2* was absent in senescent leaves and showed reduced expression in seeds at 15 days post-fertilization but was highly expressed in other tissues. In contrast, *HvGATA26* displayed elevated expression in senescent leaves compared to other tissues, implicating its potential role in regulating leaf senescence during seedling development.

To further explore the expression patterns of *HvGATA* genes in barley under abiotic stress, expression profiles were analyzed to characterize their responses to salt and drought treatments. The results revealed that under salt stress, *HvGATA1* and *HvGATA25* expression slightly decreased (**Figures 6B, C**), while *HvGATA19* and *HvGATA21* expression increased. Under normal and drought conditions, *HvGATA1* and *HvGATA19* expression declined, whereas *HvGATA21* and *HvGATA25* expression was up-regulated. A notable exception occurred under the SBC073D drought treatment, where *HvGATA1*, *HvGATA21*, and *HvGATA25* expression decreased, contrasting with the elevated expression of the *HvGATA19* gene.

### 3.6 *HvGATA* genes expression under salt and drought stress by qRT-PCR

**Figure 7:**
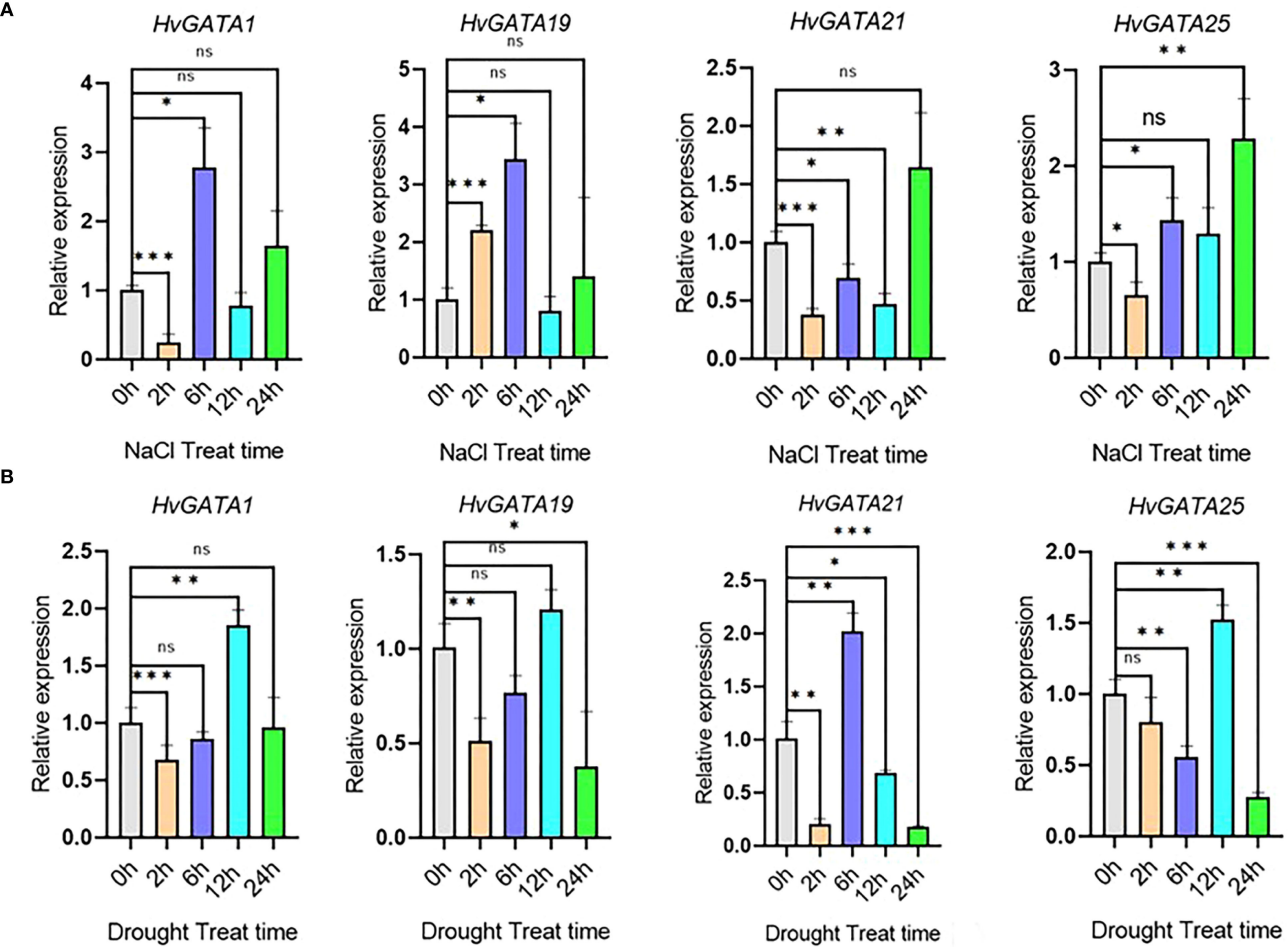
Expression patterns of *GATA* genes in barley. **(A)** Expression analysis of *GATA* genes in barley salt stress. **(B)** Expression analysis of *GATA* genes in barley drought stress. Values represent the average and standard deviation of three biological replicates. ns indicates not statistically significant, *indicates P < 0.05, **indicates P < 0.01, ***indicates P < 0.001.

In this study, qRT-PCR was used to detect the expression changes of *HvGATAs* under salt stress and drought stress conditions. Based on the expression profile of barley treated with salt and drought, four genes with high expression levels and significant changes in expression levels under salt and drought treatments (*HvGATA1*, *HvGATA19*, *HvGATA21*, and *HvGATA25*) were selected for qRT-PCR analysis. The results showed (**Figure 7A**, **Supplementary Table S9**), that under salt stress, the *HvGATA1*, *HvGATA21*, and *HvGATA25* genes were significantly down-regulated at 2 h, reaching approximately 4.17 times, 2.63 times, and 1.51 times, respectively, while the *HvGATA19* gene was significantly up-regulated by approximately 2.21 times. The *HvGATA1*, *HvGATA19*, and *HvGATA25* genes were significantly up-regulated at 6 h under salt treatment, reaching 2.78 times, 3.15 times, and 1.44 times, respectively. Furthermore, the *HvGATA21* gene was significantly down-regulated by approximately 2 times at 12 h under salt treatment. However, with the increase of salt treatment time, the *HvGATA1*, *HvGATA19*, *HvGATA21*, and *HvGATA25* genes were up-regulated by 1.64 times, 1.40 times, 1.65 times, and 2.29 times, respectively, at 24 h under salt treatment. However, significant differences emerged in the *HvGATA25* gene. The results show that these genes respond to salt stress.

Under drought stress (**Figure 7B**), with the increase of 20% PEG solution treatment time, the *HvGATA1*, *HvGATA19*, and *HvGATA21* genes were significantly down-regulated at 2 h, reaching approximately 1.47 times, 1.96 times, and 4.67 times, respectively. *HvGATA21* gene was significantly up-regulated by 2.02 times at 6 h, and the *HvGATA1* and *HvGATA25* genes were significantly up-regulated at 12 h, reaching 1.86 times and 1.52 times, respectively. At 24 h of PEG treatment, the *HvGATA19*, *HvGATA21*, and *HvGATA25* genes were significantly down-regulated, among which the expression level of *HvGATA21* gene was down-regulated by approximately 5.6 times.

### 3.7 Structural prediction and protein interaction correlation analysis of HvGATA transcription factors

Secondary structure prediction of 27 barley GATA proteins was performed using the SOPMA online tool (**Table 1**). The results demonstrate that all 27 HvGATA proteins contain α-helices, β-turns, extended strands, and random coils in their secondary structures. Random coils constitute the predominant structural component among these proteins, with HvGATA5 protein exhibiting the highest proportion (91.24%) and HvGATA16 protein the lowest (40.93%). Within subfamily IV, HvGATA6 and HvGATA16 proteins displayed notably higher α-helix contents of 40.77% and 43.41%, respectively. This elevated α-helical composition may contribute to conformational stability in these proteins.

**Table 1:** Secondary structure analysis of barley GATA transcription factors.

Gene name	Alpha helix (%)	Beta turn (%)	Random coil (%)	Extended strand (%)	Protein Secondary Structure Schematic
*HvGATA1*	32.26	1.28	63.23	3.23	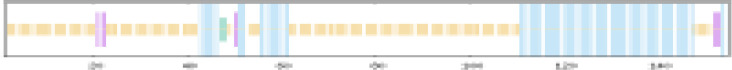
*HvGATA2*	12.22	1.39	83.89	2.50	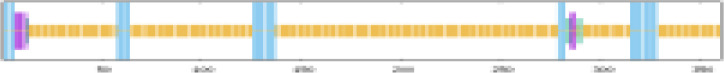
*HvGATA3*	10.74	2.76	82.82	3.68	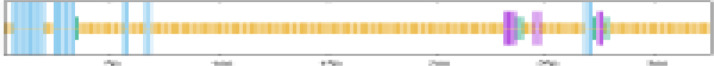
*HvGATA4*	18.04	1.06	79.58	1.33	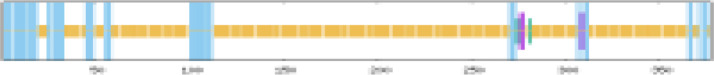
*HvGATA5*	1.84	1.84	91.24	5.07	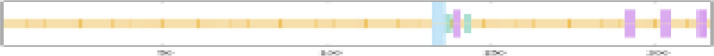
*HvGATA6*	40.77	3.23	45.03	10.97	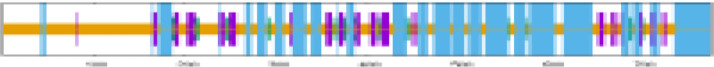
*HvGATA7*	16.01	1.16	80.28	2.55	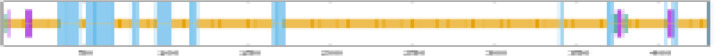
*HvGATA8*	18.14	1.36	74.60	5.90	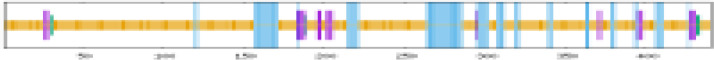
*HvGATA9*	23.03	1.10	68.20	7.68	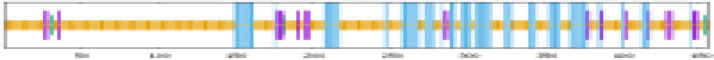
*HvGATA10*	7.33	0.86	88.79	3.02	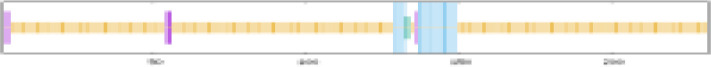
*HvGATA11*	8.42	1.52	86.73	3.32	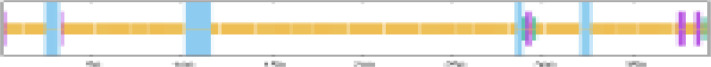
*HvGATA12*	17.68	2.21	75.69	4.42	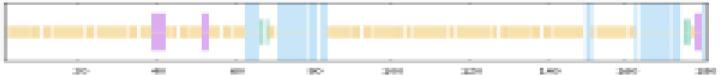
*HvGATA13*	16.46	0.95	79.75	2.85	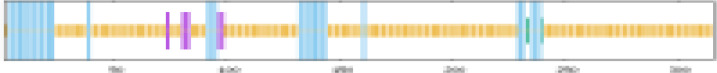
*HvGATA14*	16.85	1.87	75.28	5.99	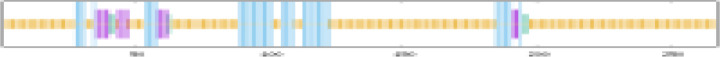
*HvGATA15*	7.69	1.14	89.74	1.42	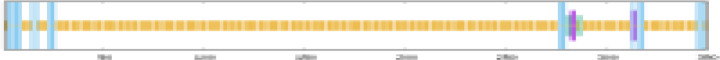
*HvGATA16*	43.41	3.71	40.93	11.95	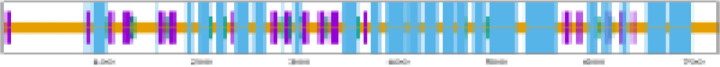
*HvGATA17*	22.18	2.42	73.39	2.02	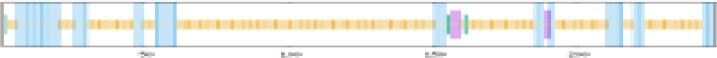
*HvGATA18*	14.85	1.12	81.79	2.24	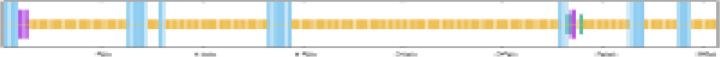
*HvGATA19*	10.89	1.43	86.25	1.43	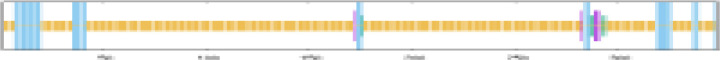
*HvGATA20*	6.95	1.35	89.46	2.24	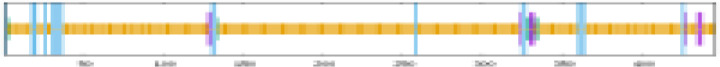
*HvGATA21*	21.60	2.47	70.99	2.47	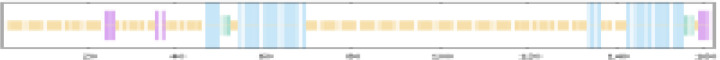
*HvGATA22*	21.96	1.78	69.14	7.12	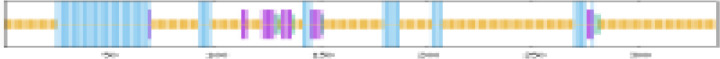
*HvGATA23*	15.25	1.17	80.06	3.52	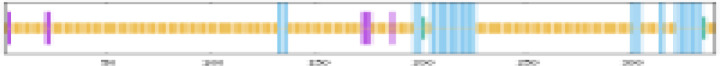
*HvGATA24*	19.01	1.49	76.79	2.72	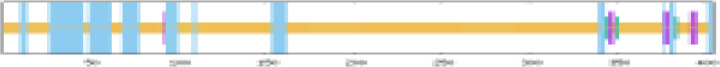
*HvGATA25*	9.88	1.23	86.42	2.47	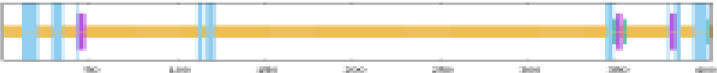
*HvGATA26*	15.00	1.11	81.39	2.50	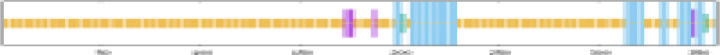
*HvGATA27*	15.44	0.74	78.68	5.15	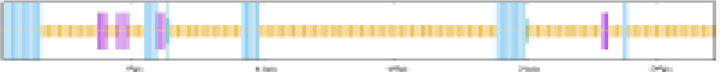

Protein Secondary Structure Schematic: Blue indicates α-helix; Green indicates β-turn; purple indicates extended strand; yellow indicates random coil.

**Figure 8:**
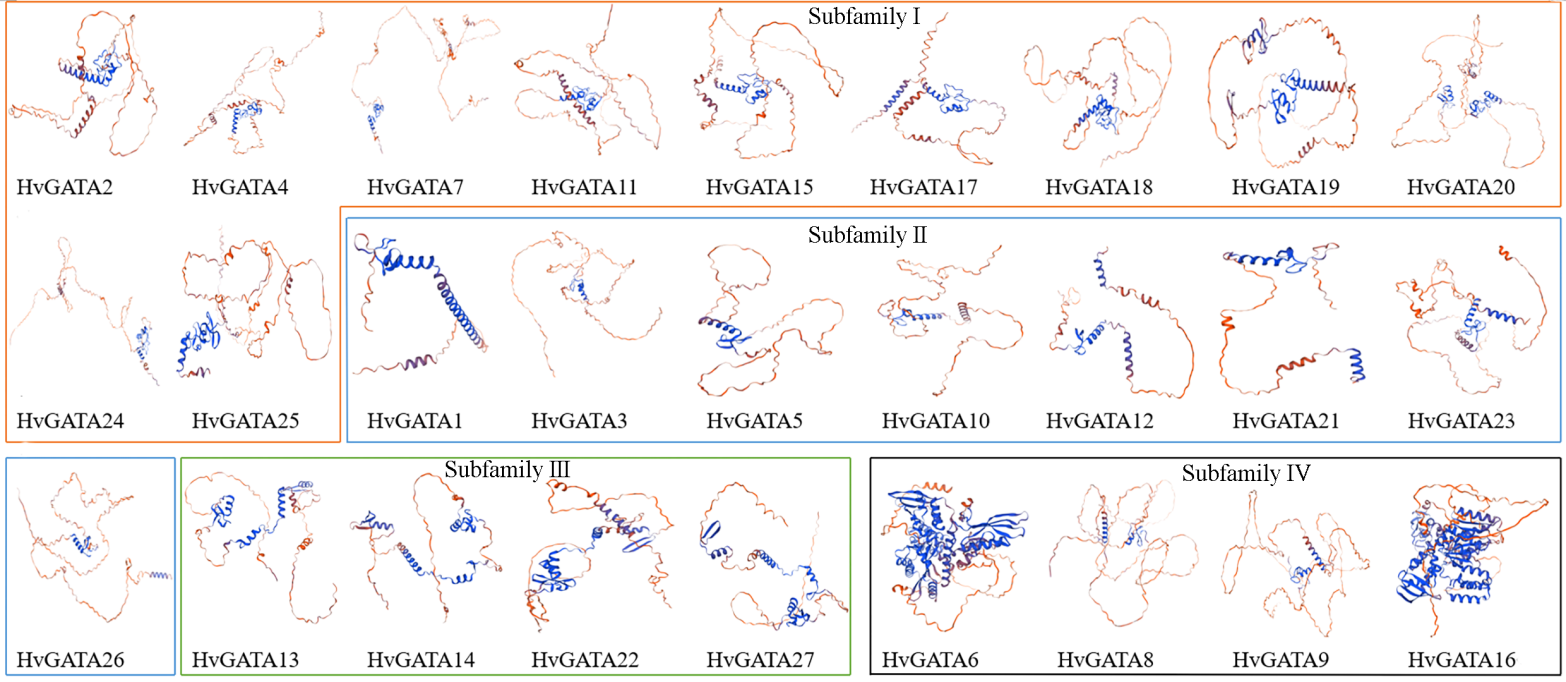
Prediction of tertiary structure of HvGATA family proteins. The distinctively colored boxes in the figure denote the tertiary structures of HvGATA proteins from different subfamilies, with orange, blue, green, and black representing subfamilies I-IV, respectively.

Tertiary structure prediction of barley GATA proteins revealed that these proteins adopt spatial conformations consisting primarily of α-helices and random coils (**Figure 8**). Comparative analysis demonstrated moderate structural complexity with significant variation in overall structural similarity. Notably, proteins within the same subfamily exhibited higher structural conservation corresponding to their phylogenetic relationships. For instance, in subfamily III, HvGATA13, HvGATA14, HvGATA22, and HvGATA27 proteins displayed strikingly similar tertiary architectures, suggesting evolutionary conservation of structural features within conserved clades.

**Figure 9:**
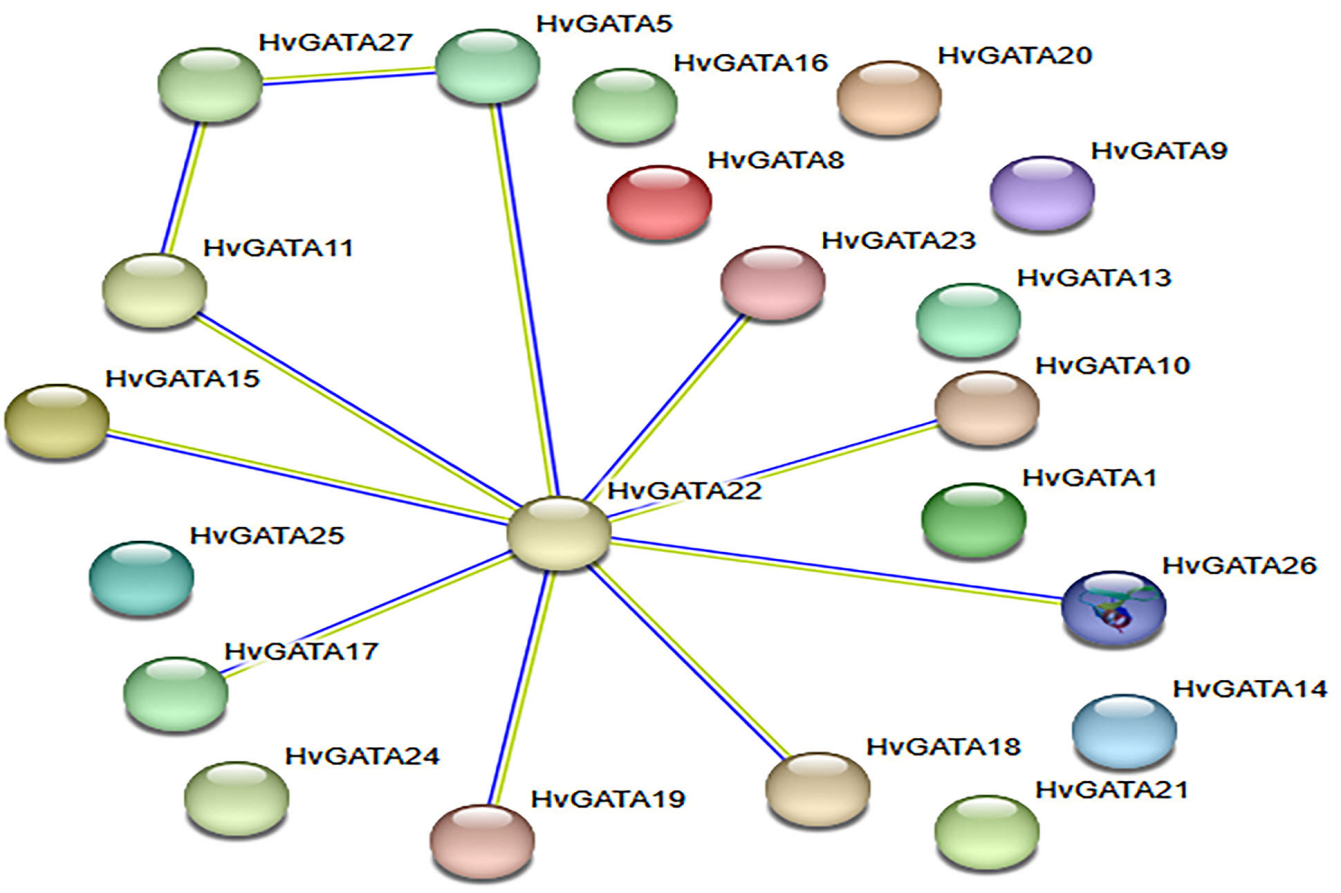
HvGATA protein-protein interaction network.

Protein interaction network analysis of barley GATA transcription factors (**Figure 9**) revealed that 10 GATA proteins (HvGATA5, HvGATA10, HvGATA11, HvGATA15, HvGATA17, HvGATA18, HvGATA19, HvGATA23, HvGATA26, and HvGATA27) form a densely interconnected cluster. This topological configuration suggests potential functional homology among these proteins, indicating their cooperative regulation in physiological and developmental processes. Conversely, 17 GATA proteins (HvGATA1-4, HvGATA6-9, HvGATA12-14, HvGATA16, HvGATA20-22, and HvGATA24-25) showed no detectable interactions, revealing significant functional diversification within this transcription factor family.

## 4 Discussion

### 4.1 Conservation of *HvGATA* genes during evolution

GATA transcription factors are a class of DNA-binding proteins ubiquitous in eukaryotes, playing pivotal roles in plant biological processes such as chlorophyll biosynthesis, photomorphogenesis, and stress adaptation. In this study, 27 *HvGATA* genes were systematically identified from the barley genome through bioinformatic analysis. Among these, 21 HvGATA proteins harbor the canonical CX_2_CX_18_CX_2_C zinc finger domain, while the remaining six possess a CX_2_CX_20_CX_2_C zinc finger domain. This domain architecture aligns with reported *GATA* families in *Arabidopsis* (Kim et al., 2021), rice (He et al., 2018), and *Brassica napus* (Zhu et al., 2020), suggesting evolutionary conservation. Notably, the variation in the number of amino acid residues (18 or 20) within the CX_2_CX_18-20_CX_2_C zinc finger domains of some GATA proteins may result from structural deletions or evolutionary divergence. Substantial variation was observed in HvGATA protein properties, including amino acid length (ranging from 155 to 775 aa), molecular weight (17343.27 Da-88608.50 Da), and isoelectric point (pI 4.66-10.22), potentially reflecting functional diversification. Prediction analysis of barley GATA protein structures and interaction relationships demonstrated that secondary structures were predominantly composed of random coils. Overall similarity of tertiary structures existed with differences, but the same subfamily exhibited higher similarity, indicating that members within the same subfamily possess high structural homology during evolution. Protein interaction correlation analysis also indicated functional similarity among family members, suggesting cooperative regulatory functions in certain plant growth and developmental processes. Phylogenetic clustering, supported by conserved motif and gene structure analyses, classified barley *GATA* members into four subfamilies, consistent with classifications in *Arabidopsis* (Reyes et al., 2004), rice (Reyes et al., 2004), and *Sorghum bicolor* (Yao et al., 2023). Functional predictions for barley *GATA* genes were inferred based on orthologous subfamily functions in *Arabidopsis*. Notably, members within the same subfamily exhibited highly similar motif compositions and exon-intron architectures, indicating strong evolutionary conservation and potential functional redundancy. Conversely, structural divergence across subfamilies likely arose from lineage-specific evolutionary pressures, such as domain shuffling or selective gene loss.

In higher plants, the *GATA* gene family exhibits significant variation in member numbers across species, even among closely related taxa. For instance, wheat harbors 79 *GATA* members (Buzby et al., 1990), compared to 30 in *Arabidopsis* (dicot) (Manfield et al., 2007) and 28 in rice (monocot) (Gupta et al., 2017). Such divergence likely reflects lineage-specific evolutionary trajectories, where gene duplication events-key drivers of family expansion play pivotal roles (Feng et al., 2022). In barley, we identified seven segmental duplication events among 27 *HvGATA* genes, with no tandem duplications observed. Notably, two duplication pairs localized to Subfamilies I and II, suggesting subfunctionalization or neofunctionalization within these clades. Synteny analysis further revealed stronger conservation between barley and rice than with *Arabidopsis*, corroborating closer phylogenetic relationships within the poaceae family.

### 4.2 *HvGATA* genes are involved in hormone signaling and abiotic stress response

*Cis*-regulatory elements are non-coding DNA sequences within gene promoters that orchestrate transcriptional regulation. Analyzing these elements in *HvGATA* promoters provides critical insights into their functional diversification in barley. Previous studies demonstrate that plant GATA transcription factors mediate light signaling by binding to GATA motifs in photoresponsive gene promoters (Cannon et al., 2004), exemplified by *GATA2*’s dual role in modulating light and brassinosteroid signaling pathways (Luo et al., 2010). This functional interplay suggests that *GATA* genes may both regulate and be regulated by light-responsive genes, underscoring the importance of *HvGATA* promoter analysis in barley. Plant GATA factors are also implicated in stress adaptation, developmental regulation, and hormone signaling (Manfield et al., 2007; Richter et al., 2013), consistent with our findings in the barley *HvGATA* family. Promoter analysis revealed an enrichment of stress-responsive *cis*-elements (e.g., drought-inducible MBS MYC) and hormone-related motifs (ABRE for abscisic acid, GARE for gibberellins, TGA-element for auxin, and CGTCA-motif for jasmonate) in H*vGATA* promoters. Additionally, specific *HvGATA* genes harbor elements associated with meristem expression (e.g., CAT-box), endosperm specificity (e.g., GCN4-motif), and hypoxia response (e.g., GC-motif), indicating their potential roles in diverse physiological processes. However, while these *cis*-element profiles suggest environmental sensitivity and multifunctional regulation, direct experimental evidence is required to confirm their binding efficacy and regulatory polarity (activation or repression) under specific conditions.

### 4.3 *HvGATA* genes play an important role in the response mechanism of barley salt and drought stress

Gene expression patterns across tissues often reflect functional specialization. For instance, *OsGATA23a* in rice exhibits broad stress responsiveness, with significant up-regulation under salt and drought conditions (Gupta et al., 2017), underscoring the conserved regulatory role of GATA transcription factors in stress signaling. In barley, tissue-specific expression profiling of four *HvGATA* genes revealed conserved expression patterns within subfamilies, indicative of functional conservation. qRT-PCR analysis further demonstrated that *HvGATA* genes are dynamically regulated under abiotic stress. Under salt stress, at 2 h, only *HvGATA19* was significantly up-regulated, while the other three genes were significantly down-regulated. *HvGATA1* and *HvGATA19* were transiently up-regulated at 6 h post-treatment, whereas *HvGATA21* and *HvGATA25* peaked at 12 h. Conversely, drought stress triggered pronounced down-regulation of *HvGATA1*, *HvGATA*19, and *HvGATA21* at 2h. At 12 h, *HvGATA1* and *HvGATA25* genes were significantly up-regulated. The *HvGATA19*, *HvGATA21*, and *HvGATA25* genes were significantly down-regulated by approximately 2.63 times, 5.56 times, and 3.57 times, respectively, at 24 h. These temporal and stress-specific expression dynamics suggest functional diversification within the *HvGATA* family, enabling coordinated regulation of developmental processes and stress adaptation.

## 5 Conclusion

In this study, 27 *GATA* genes were identified in the barley genome using bioinformatics approaches and classified into four subfamilies. Members within the same *GATA* subfamily exhibit higher sequence similarity and conservation, suggesting potential functional similarities. Analysis of promoter regions indicated that the cis-acting elements associated with *HvGATA* genes may be subject to complex regulation, particularly elements responsive to hormone signaling pathways and abiotic stress. Furthermore, the expression patterns of *HvGATA1*, *HvGATA19*, *HvGATA21*, and *HvGATA25* genes differed following salt and drought stress treatments applied over different time courses, indicating their potential involvement in barley’s salt and drought tolerance mechanisms.

## Data Availability

The datasets presented in this study can be found in online repositories. The names of the repository/repositories and accession number(s) can be found in the article/**Supplementary Material**.

## References

[B1] Altschul S. F. Madden T. L. Schaffer A. A. Zhang J. Zhang Z. Miller W.. (1997). Gapped BLAST and PSI-BLAST: a new generation of protein database search programs. Nucleic Acids Res. 25, 3389–3402. doi: 10.1093/nar/25.17.3389, PMID: 9254694 PMC146917

[B2] Bailey T. L. Boden M. Buske F. A. Frith M. Grant C. E. Clementi L.. (2009). MEME SUITE: tools for motif discovery and searching. Nucleic Acids Res. 37, W202–W208. doi: 10.1093/nar/gkp335, PMID: 19458158 PMC2703892

[B3] Behringer C. Schwechheimer C. (2015). B-GATA transcription factors-insights into their structure, regulation, and role in plant development. Front. Plant Sci. 6. doi: 10.3389/fpls.2015.00090, PMID: 25755661 PMC4337238

[B4] Bi Y. M. Zhang Y. Signorelli T. Zhao R. Zhu T. Rothstein S. (2005). Genetic analysis of Arabidopsis GATA transcription factor gene family reveals a nitrate-inducible member important for chlorophyll synthesis and glucose sensitivity. Plant J. 44, 680–692. doi: 10.1111/j.1365-313X.2005.02568.x, PMID: 16262716

[B5] Buzby J. S. Yamada T. Tobin E. M. (1990). A light-regulated DNA-binding activity interacts with a conserved region of a Lemna gibba rbcS promoter. Plant Cell. 2, 805–814. doi: 10.1105/tpc.2.8.805, PMID: 2152129 PMC159932

[B6] Cannon S. B. Mitra A. Baumgarten A. (2004). The roles of segmental and tandem gene duplication in the evolution of large gene families in *Arabidopsis thaliana* . BMC Plant Biol. 4, 10. doi: 10.1186/1471-2229-4-10, PMID: 15171794 PMC446195

[B7] Chen C. Chen H. Zhang Y. Thomas H. R. Frank M. H. He Y.. (2020). TBtools: an integrative toolkit developed for interactive analyses of big biological data. Mol. Plant 13, 1194–1202. doi: 10.1016/j.molp.2020.06.009, PMID: 32585190

[B8] Daniel-Vedele F. Caboche M. (1993). A tobacco cDNA clone encoding a GATA-1 zinc finger protein homologous to regulators of nitrogen metabolism in fungi. Mol. Gen. Genet. 240, 365–373. doi: 10.1007/BF00280388, PMID: 8413186

[B9] Darigh F. Iranbakhsh A. Oraghi Ardebili Z. Ebadi M. Hassanpour H. (2022). Simulated microgravity contributed to modification of callogenesis performance and secondary metabolite production in *Cannabis Indica* . Plant Physiol. Biochem. 186, 157–168. doi: 10.1016/j.plaphy.2022.07.012, PMID: 35849945

[B10] Dubos C. Stracke R. Grotewold E. Weisshaar B. Martin C. Lepiniec L.. (2010). MYB transcription factors in *Arabidopsis* . Trends Plant Sci. 15, 573–581. doi: 10.1016/j.tplants.2010.06.005, PMID: 20674465

[B11] Feng X. Yu Q. Zeng J. He X. Liu W. (2022). Genome-wide identification and characterization of GATA family genes in wheat. BMC Plant Biol. 22, 372. doi: 10.1186/s12870-022-03733-3, PMID: 35896980 PMC9327314

[B12] Finn R. D. Clements J. Eddy S. R. (2011). HMMER web server: interactive sequence similarity searching. Nucleic Acids Res. 39, W29–W37. doi: 10.1093/nar/gkr367, PMID: 21593126 PMC3125773

[B13] Finn R. D. Tate J. Mistry J. Coggill P. C. Sammut S. J. Hotz H. R.. (2008). The Pfam protein families database. Nucleic Acids Res. 36, D281–D288. doi: 10.1093/nar/gkp985, PMID: 18039703 PMC2238907

[B14] Geng L. Li M. Zhang G. Ye L. Z. (2022). Barley: a potential cereal for producing healthy and functional foods. Food Qual. safety. 6, 142–154. doi: 10.1093/fqsafe/fyac012

[B15] Guo A. Y. Chen G. G. Zhang H. Zhu Q. H. Liu X. C. Zhong Y. F.. (2008). Plant TFDB: a comprehensive plant transcription factor database. Nucleic Acids Res. 36, D966–D969. doi: 10.1093/nar/gkm841, PMID: 17933783 PMC2238823

[B16] Gupta P. Nutan K. K. Singla-Pareek S. L. Pareek A. (2017). Abiotic stresses cause differential regulation of alternative splice forms of GATA transcription factor in rice. Front. Plant Sci. 8. doi: 10.3389/fpls.2017.01944, PMID: 29181013 PMC5693882

[B17] He P. Wang X. Zhang X. Jiang Y. Tian W. Zhang X.. (2018). Short and narrow flag leaf1, a GATA zinc finger domain-containing protein, regulates flag leaf size in rice (*Oryza sativa*). BMC Plant Biol. 18, 273–273. doi: 10.1186/s12870-018-1452-9, PMID: 30413183 PMC6230254

[B18] Huang X. Y. Chao D. Y. Gao J. P. Zhu M. Z. Shi M. Lin H. X. (2009). A previously unknown zinc finger protein, DST, regulates drought and salt tolerance in rice via stomatal aperture control. Genes Dev. 23, 1805–1817. doi: 10.1101/gad.1812409, PMID: 19651988 PMC2720257

[B19] Hudson D. Guevara D. R. Hand A. J. Xu Z. Hao L. Chen X.. (2013). Rice cytokinin GATA transcription Factor1 regulates chloroplast development and plant architecture. Plant Physiol. 162, 132–144. doi: 10.1104/pp.113.217265, PMID: 23548780 PMC3641198

[B20] Hudson D. Guevara D. Yaish M. W. Hannam C. Long N. Clarke J. D.. (2011). *GNC* and *CGA1* modulate chlorophyll biosynthesis and glutamate synthase (*GLU1/Fd-GOGAT*) expression in *Arabidopsis* . PloS One 6, e26765. doi: 10.1371/journal.pone.0026765, PMID: 22102866 PMC3213100

[B21] Jiang J. Ma S. Ye N. Jiang M. Cao J. Zhang J. (2017). WRKY transcription factors in plant responses to stresses. J. Integr. Plant Biol. 59, 86–101. doi: 10.1111/jipb.12513, PMID: 27995748

[B22] Kim M. (2024). Comparative analysis of amino acid sequence level in plant GATA transcription factors. Sci. Rep. 14, 29786. doi: 10.1038/s41598-024-81159-7, PMID: 39616200 PMC11608367

[B23] Kim M. Xi H. Park S. Yun Y. Park J. (2021). Genome-wide comparative analyses of GATA transcription factors among seven *Populus* genomes. Sci. Rep. 11, 16578. doi: 10.1038/s41598-021-95940-5, PMID: 34400697 PMC8367991

[B24] Krzywinski M. Schein J. Birol I. Connors J. Gascoyne R. Horsman D.. (2009). Circos: an information aesthetic for comparative genomics. Genome Res. 19, 1639–1645. doi: 10.1101/gr.092759.109, PMID: 19541911 PMC2752132

[B25] Larkin M. A. Blackshields G. Brown N. P. Chenna R. McGettigan P. A. McWilliam H.. (2007). Clustal W and clustal X version 2.0. Bioinformatics 23, 2947–2948. doi: 10.1093/bioinformatics/btm404, PMID: 17846036

[B26] Letunic I. Bork P. (2018). 20 years of the SMART protein domain annotation resource. Nucleic Acids Res. 46, D493–D496. doi: 10.1093/nar/gkx922, PMID: 29040681 PMC5753352

[B27] Liu H. Yuan L. Guo W. Wu W. (2022). Transcription factor TERF1 promotes seed germination under osmotic conditions by activating gibberellin acid signaling. Plant Sci. 322, 111350. doi: 10.1016/j.plantsci.2022.111350, PMID: 35709980

[B28] Lowry J. A. Atchley W. R. (2000). Molecular evolution of the GATA family of transcription factors: conservation within the DNA-binding domain. J. Mol. Evol. 50, 103–115. doi: 10.1007/s002399910012, PMID: 10684344

[B29] Luo X. M. Lin W. H. Zhu S. Zhu J. Y. Sun Y. Fan X. Y.. (2010). Integration of light- and brassinosteroid-signaling pathways by a GATA transcription factor in *Arabidopsis* . Dev. Cell. 19, 872–883. doi: 10.1016/j.devcel.2010.10.023, PMID: 21145502 PMC3022420

[B30] Manfield I. W. Devlin P. F. Jen C. H. Westhead D. R. Gilmartin P. M. (2007). Conservation, convergence, and divergence of light-responsive, circadian-regulated, and tissue-specific expression patterns during evolution of the Arabidopsis GATA gene family. Plant Physiol. 143, 941–958. doi: 10.1104/pp.106.090761, PMID: 17208962 PMC1803723

[B31] Reyes J. C. Muro-Pastor M. I. Florencio F. J. (2004). The GATA family of transcription factors in Arabidopsis and rice. Plant Physiol. 134, 1718–1732. doi: 10.1104/pp.103.037788, PMID: 15084732 PMC419845

[B32] Richter R. Behringer C. Zourelidou M. Schwechheimer C. (2013). Convergence of auxin and gibberellin signaling on the regulation of the GATA transcription factors *GNC* and *GNL* in *Arabidopsis thaliana* . Proc. Natl. Acad. Sci. 110, 13192–13197. doi: 10.1073/pnas.1304250110, PMID: 23878229 PMC3740866

[B33] Rueda-Lopez M. Canas R. A. Canales J. Canovas F. M. Avila C. (2015). The overexpression of the pine transcription factor PpDof5 in *Arabidopsis* leads to increased lignin content and affects carbon and nitrogen metabolism. Physiol. Plant 155, 369–383. doi: 10.1111/ppl.12381, PMID: 26333592

[B34] Rushton P. J. Somssich I. E. Ringler P. Shen Q. J. (2010). WRKY transcription factors. Trends Plant Sci. 15, 247–258. doi: 10.1016/j.tplants.2010.02.006, PMID: 20304701

[B35] Scazzocchio C. (2000). The fungal GATA factors. Curr. Opin. Microbiol. 3, 126–131. doi: 10.1016/s1369-5274(00)00063-1, PMID: 10745000

[B36] Stevanovic M. Stanisavljevic Ninkovic D. Mojsin M. Drakulic D. Schwirtlich M. (2022). Interplay of SOX transcription factors and microRNAs in the brain under physiological and pathological conditions. Neural Regener. Res. 17, 2325–2334. doi: 10.4103/1673-5374.338990, PMID: 35535866 PMC9120710

[B37] Tamura K. Stecher G. Kumar S. (2021). MEGA11: molecular evolutionary genetics analysis version 11. Mol. Biol. Evol. 38, 3022–3027. doi: 10.1093/molbev/msab120, PMID: 33892491 PMC8233496

[B38] Teakle G. R. Gilmartin P. M. (1998). Two forms of type IV zinc-finger motif and their kingdom-specific distribution between the flora, fauna and fungi. Trends Biochem. Sci. 23, 100–102. doi: 10.1016/s0968-0004(98)01174-8, PMID: 9581501

[B39] Wang Y. Tang H. Debarry J. D. Tan X. Li J. Wang X.. (2012). MCScanX: a toolkit for detection and evolutionary analysis of gene synteny and collinearity. Nucleic Acids Res. 40, e49. doi: 10.1093/nar/gkr1293, PMID: 22217600 PMC3326336

[B40] Wang R. Zhao P. Kong N. Lu R. Pei Y. Huang C.. (2018). Genome-wide identification and characterization of the potato bHLH transcription factor family. Genes 9, 54. doi: 10.3390/genes9010054, PMID: 29361801 PMC5793205

[B41] Wang F. Zhu D. Huang X. Li S. Gong Y. Yao Q.. (2009). Biochemical insights on degradation of Arabidopsis DELLA proteins gained from a cell-free assay system. Plant Cell. 21, 2378–2390. doi: 10.1105/tpc.108.065433, PMID: 19717618 PMC2751948

[B42] Wei X. Li Y. Zhu X. Liu X. Ye X. Zhou M.. (2023). The GATA transcription factor TaGATA1 recruits demethylase TaELF6-A1 and enhances seed dormancy in wheat by directly regulating *TaABI5* . J. Integr. Plant Biol. 65, 1262–1276. doi: 10.1111/jipb.13437, PMID: 36534453

[B43] Yang M. Derbyshire M. K. Yamashita R. A. Marchler-Bauer A. (2020). NCBI’s conserved domain database and tools for protein domain analysis. Curr. Protoc. Bioinf. 69, e90. doi: 10.1002/cpbi.90, PMID: 31851420 PMC7378889

[B44] Yao X. Lai D. Zhou M. Ruan J. Ma C. Wu W.. (2023). Genome-wide identification, evolution and expression pattern analysis of the *GATA* gene family in *Sorghum bicolor* . Front. Plant Sci. 14. doi: 10.3389/fpls.2023.1163357, PMID: 37600205 PMC10437121

[B45] Zeng X. Ling H. Chen X. Guo S. (2019). Genome-wide identification, phylogeny and function analysis of GRAS gene family in *Dendrobium catenatum* (Orchidaceae). Gene 705, 5–15. doi: 10.1016/j.gene.2019.04.038, PMID: 30999026

[B46] Zha Q. Xi X. He Y. Yin X. Jiang A. (2022). Interaction of VvbZIP60s and VvHSP83 in response to high-temperature stress in grapes. Gene 810, 146053. doi: 10.1016/j.gene.2021.146053, PMID: 34757157

[B47] Zhan J. Thakare D. Ma C. Lloyd A. Nixon N. M. Arakaki A. M.. (2015). RNA sequencing of laser-capture microdissected compartments of the maize kernel identifies regulatory modules associated with endosperm cell differentiation. Plant Cell. 27, 513–531. doi: 10.1105/tpc.114.135657, PMID: 25783031 PMC4558669

[B48] Zheng J. Zhang Z. Tong T. Fang Y. Zhang X. (2021). Genome-wide identification of WRKY gene family and expression analysis under abiotic stress in barley. Agronomy 11, 521. doi: 10.3390/agronomy11030521

[B49] Zhu W. Guo Y. Chen Y. Wu D. Jiang L. (2020). Genome-wide identification, phylogenetic and expression pattern analysis of *GATA* family genes in Brassica napus. BMC Plant Biol. 20, 543. doi: 10.1186/s12870-020-02752-2, PMID: 33276730 PMC7716463

